# Supplementary material for: The Identification and Comparative Analysis of Non-Coding RNAs in Spores and Mycelia of Penicillium expansum
Source: J Fungi (Basel). 2023 Oct 9;9(10):999. doi: 10.3390/jof9100999 (PMC10607695; doi:10.3390/jof9100999)
Supplement: Supplementary file 1 [file jof-09-00999-s001.zip › Supplementary Figures.pdf]

# The identification and comparative analysis of non-coding RNAs in spores and mycelia of *Penicillium expansum*

Tongfei Lai [1], Qinru Yu [1], Jingjing Pan [1], Jingjing Wang [1], Zhenxing Tang [2], Xuelian Bai [1], Lue Shi [1] and Ting Zhou [1,*]

[1] College of Life and Environmental Science, Hangzhou Normal University, Hangzhou 310036, China; ; laitongfei@hznu.edu.cn (T.L.); 2021111010050@stu.hznu.edu.cn (Q.Y.); 2022111010065@stu.hznu.edu.cn (J.P.); 2022111010055@stu.hznu.cn (J.W.); baixl2012@163.com (X.B.); shilue@126.com (L.S.)

[2] School of Culinary Arts, Tourism College of Zhejiang, Hangzhou 311231, China; tangzhenxing@126.com

* Correspondence: zt20100061@hznu.edu.cn; Tel.: +86-571-28861007

**Supporting information**

**Supporting tables**

**Table S1.** The information on primer pairs for qRT-PCR validation

**Table S2.** The summary of lncRNA sequencing results of *P. expansum*

**Table S3.** The information of assembled transcripts in *P. expansum*

The green, yellow and blue zones show the information on known mRNAs, novel mRNAs and lncRNAs, respectively.

**Table S4.** The annotation of identified mRNAs in *P. expansum*

NR: Non-redundant protein sequence database; NT: Nucleotide sequence database; COG: Clusters of orthologous genes database; KEGG: Kyoto encyclopedia of genes and genomes database; Swissprot: Swiss-Prot database; NA: Non-applicable.

**Table S5.** The expression levels of all transcripts in *P. expansum*

The green, yellow and blue zones show the information on known mRNAs, novel mRNAs and lncRNAs, respectively.

**Table S6.** The differentially expressed transcripts between Pe6h and Pe18h in *P. expansum*

FDR: False discovery rate.

**Table S7.** The lncRNA family prediction in *P. expansum*

**Table S8.** The prediction of lncRNA target genes in *P. expansum*

NA: Non-applicable; +: sense; -: anti-sense; *: significance correlation.

**Table S9.** The information of predicted target genes of differentially expressed lncRNAs in *P. expansum*

NA: Non-applicable; * indicates significant correlation.

**Table S10.** Summary of small RNA sequencing results in *P. expansum*

**Table S11.** The known miRNAs identified in *P. expansum*

**Table S12.** The predicted novel miRNAs in *P. expansum*

**Table S13.** The predicted novel siRNA in *P. expansum*

**Table S14.** The expression levels of small RNAs in *P. expansum*

TPM: Transcripts per million

**Table S15.** The prediction of miRNA target genes in *P. expansum*

**Table S16.** The correlation among lncRNAs, miRNAs and mRNAs in *P. expansum*

**Table S17.** The prediction of circRNAs in *P. expansum*

**Table S18.** The source genes of differentially expressed circRNAs between Pe6h and

Pe18h in *P. expansum*

## Supporting figures and figure legends

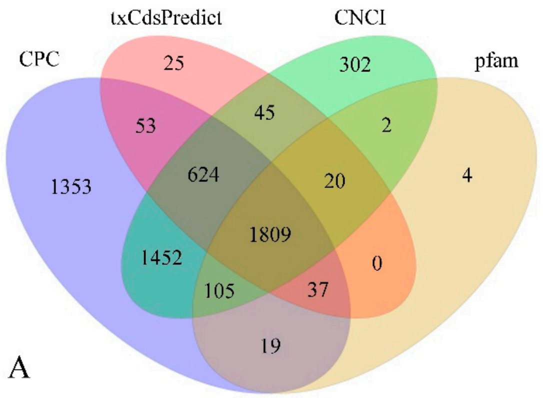


Figure S1. Venn figure of coding capacity prediction by. A: Predicted results of mRNAs; B: Predicted results of lncRNAs. Different colors represent different prediction methods including the Pfam database and three software CPC, txCdsPredict and CNCI.

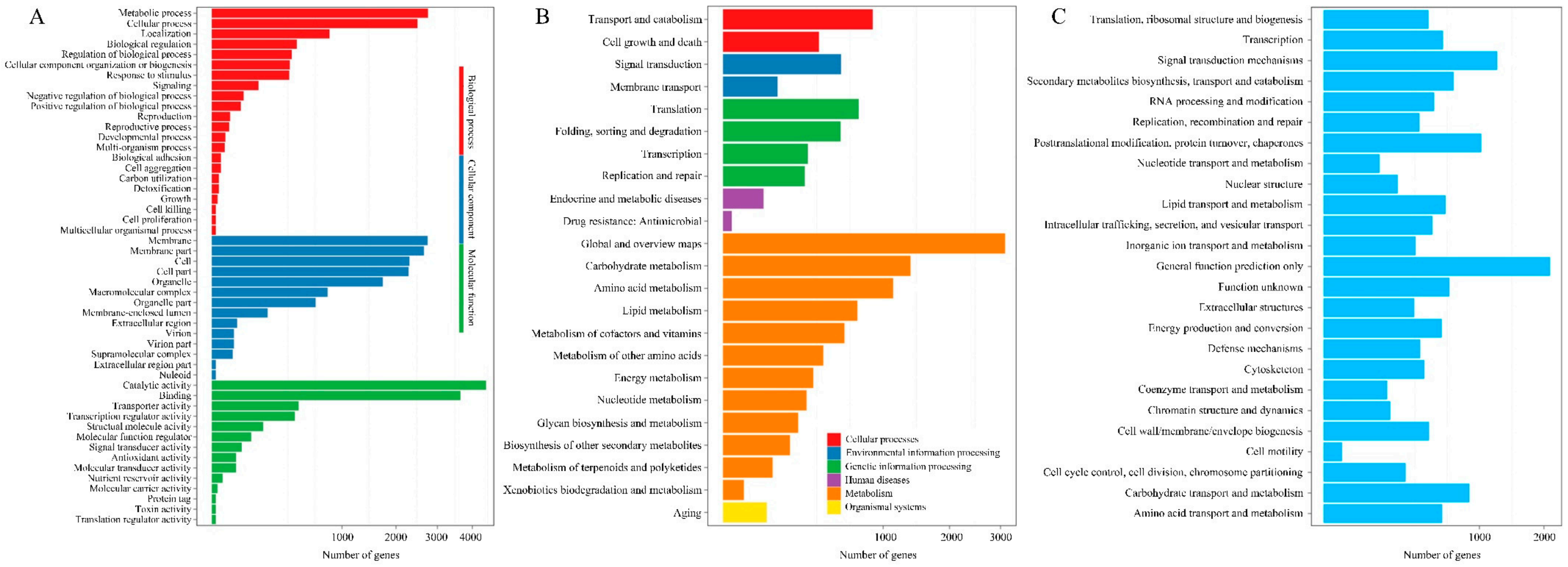


Figure S2. The mRNA genes annotation. A: GO annotation statistics; B: KEGG annotation statistics; C: COG annotation statistics. The X-axis represents the number of genes, and the Y-axis represents the GO, KEGG or COG entry. The different color indicates the different classification of GO or KEGG.

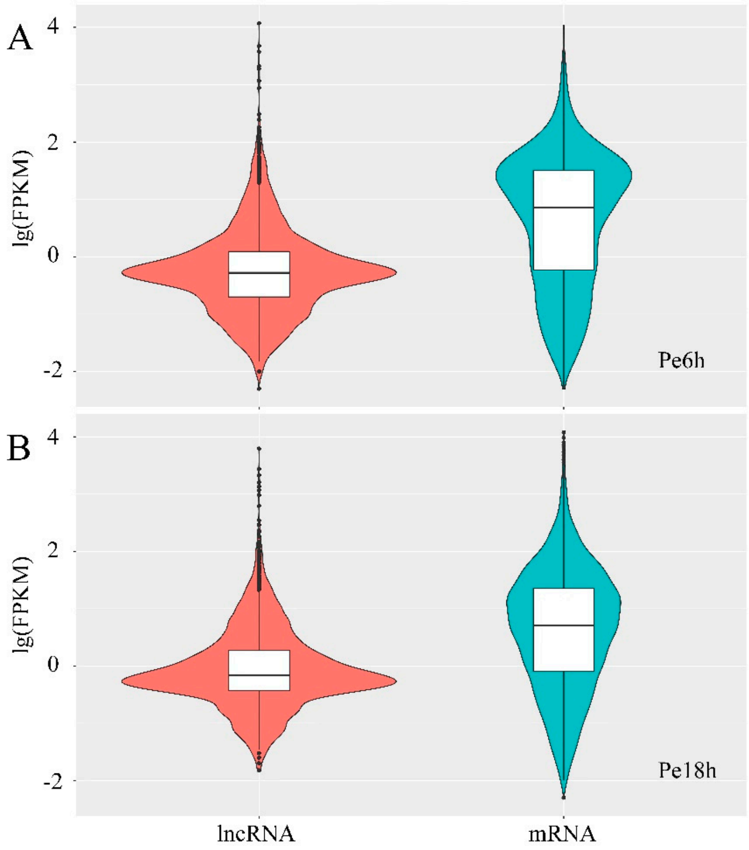


Figure S3. The expression density distribution of lncRNAs and mRNAs in Pe16h (A) and Pe18h (B). X-axis: RNA density of a certain expression, Y-axis: the value of log10 of FPKM. From the top of the figure, there is the upper quarter, the median (black thick line) and the lower quarter. The median is the number in the middle positions in the data, that is, half of the data is greater than the median (above), and the other half is less than the median (below). A quarter of the data is greater than the upper quartile, which is above the white box; another quarter of the data is less than the lower quartile, which is under the white box.

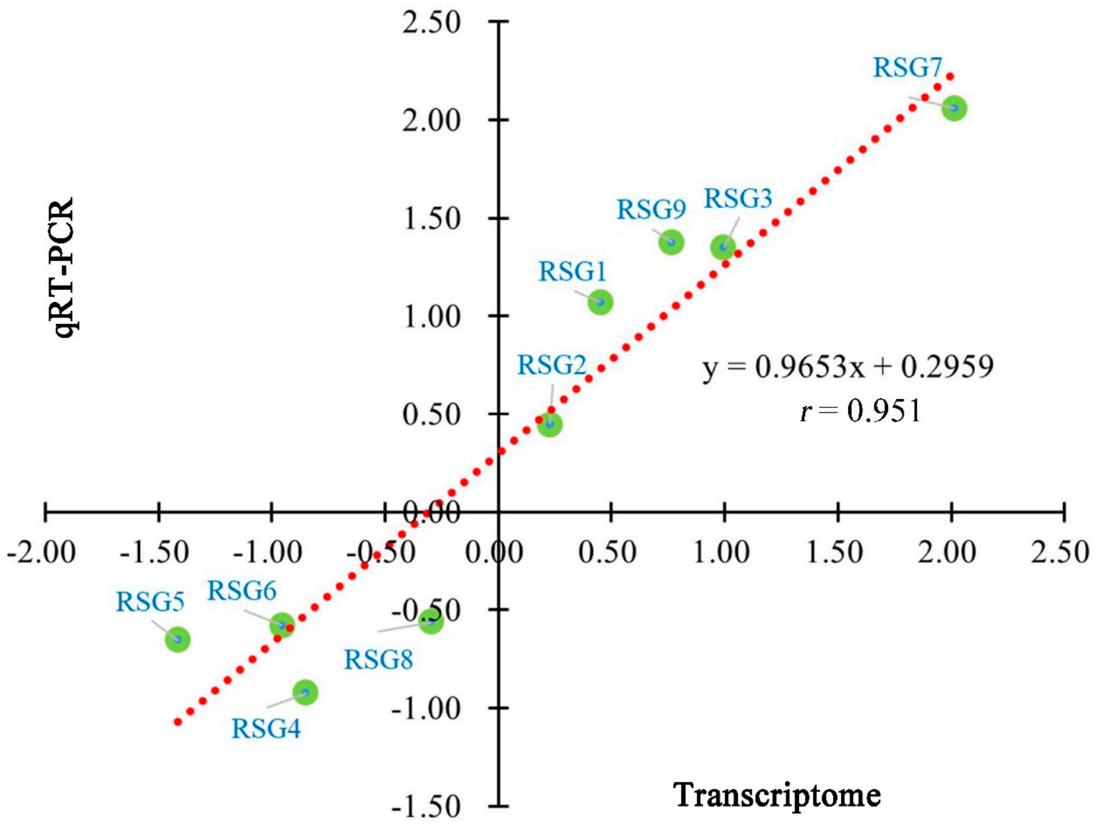


Figure S4. Regression analysis of RSGs relative expression exposed by qRT-PCR and transcriptome sequencing. The X axis indicates $log_2$Ratio(Pe18h/Pe6h) acquired from transcriptome sequencing method, and the Y axis indicates $log_2$Ratio(Pe18h/Pe6h) acquired from qRT-PCR method. The green dots represent the different RSGs and the red dotted line indicates optimum imitative straight line. The *r* indicates the correlation coefficient.

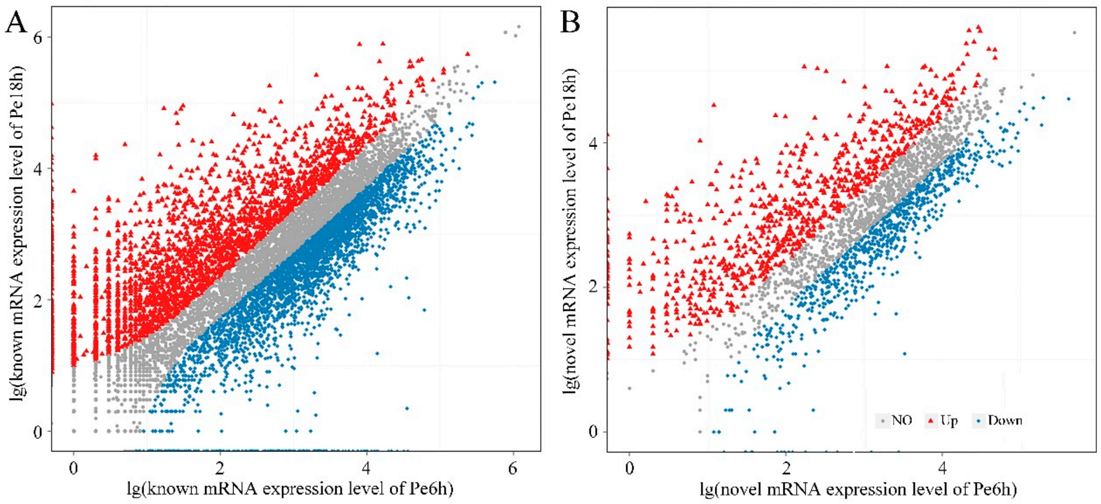


Figure S5. The scatter plot of the differentially expressed known mRNAs (A) and novel mRNAs (B) between Pe6h and Pe18h. Blue color squares represent the down-regulated genes, red color triangles represent the up-regulated genes, and grey color dots represent the no significantly different genes.

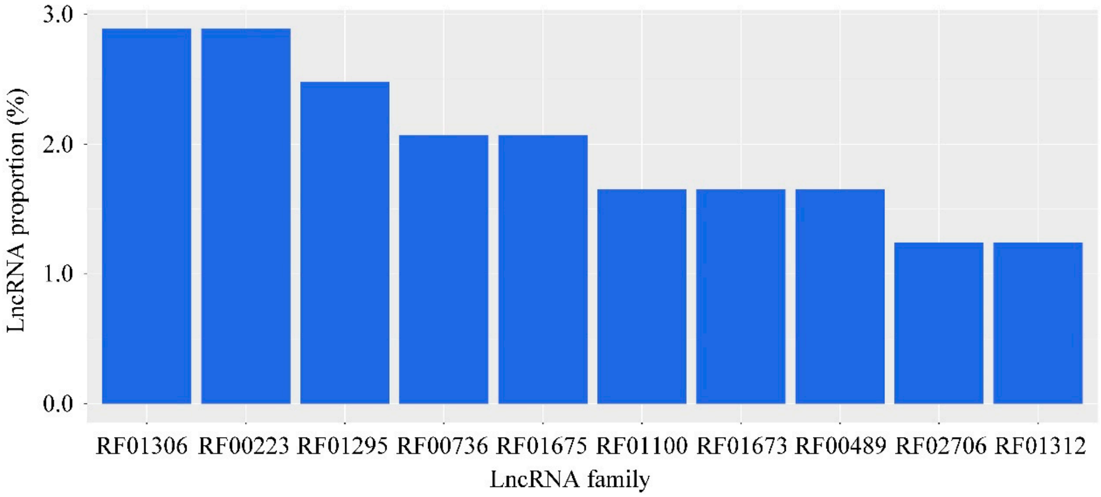


Figure S6. The lncRNA family statistical results

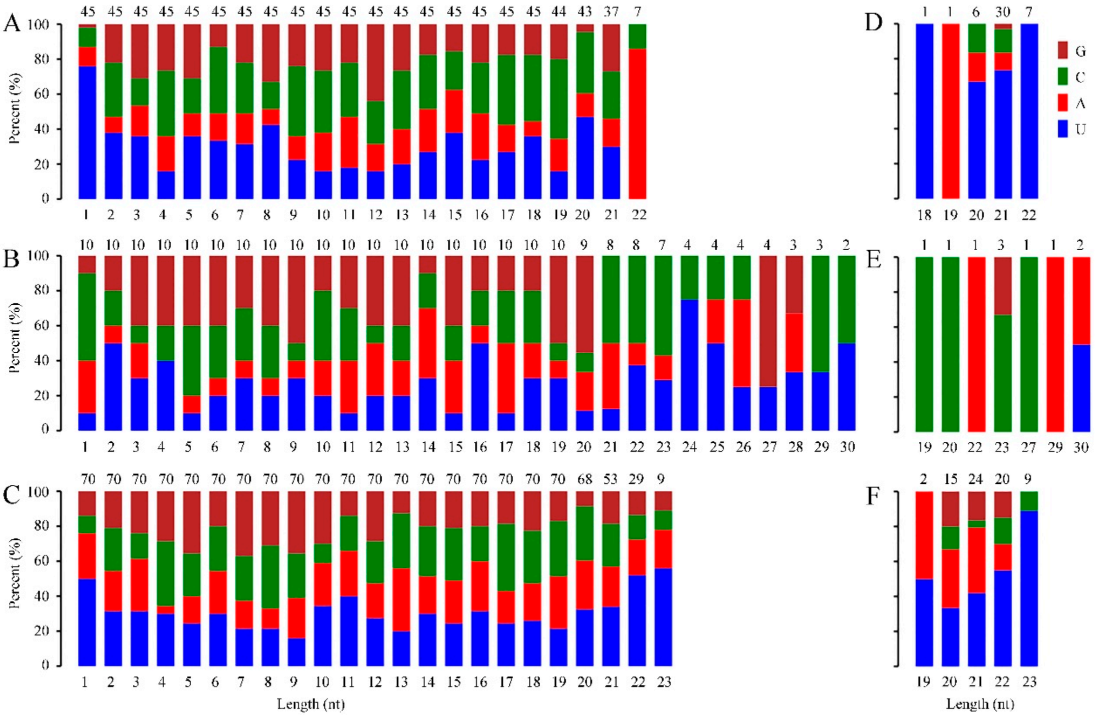


Figure S7. The base distribution of miRNAs and siRNAs in *P. expansum*. A, B and C show the base distribution of known miRNAs, novel miRNAs and novel siRNAs, respectively. X-axis represents the base position; D, E and F show the first base distribution of known miRNAs, novel miRNAs and novel siRNAs, respectively. X-axis represents the length of RNAs. The number on the bar indicates the number of RNAs, and different colors represent different bases.

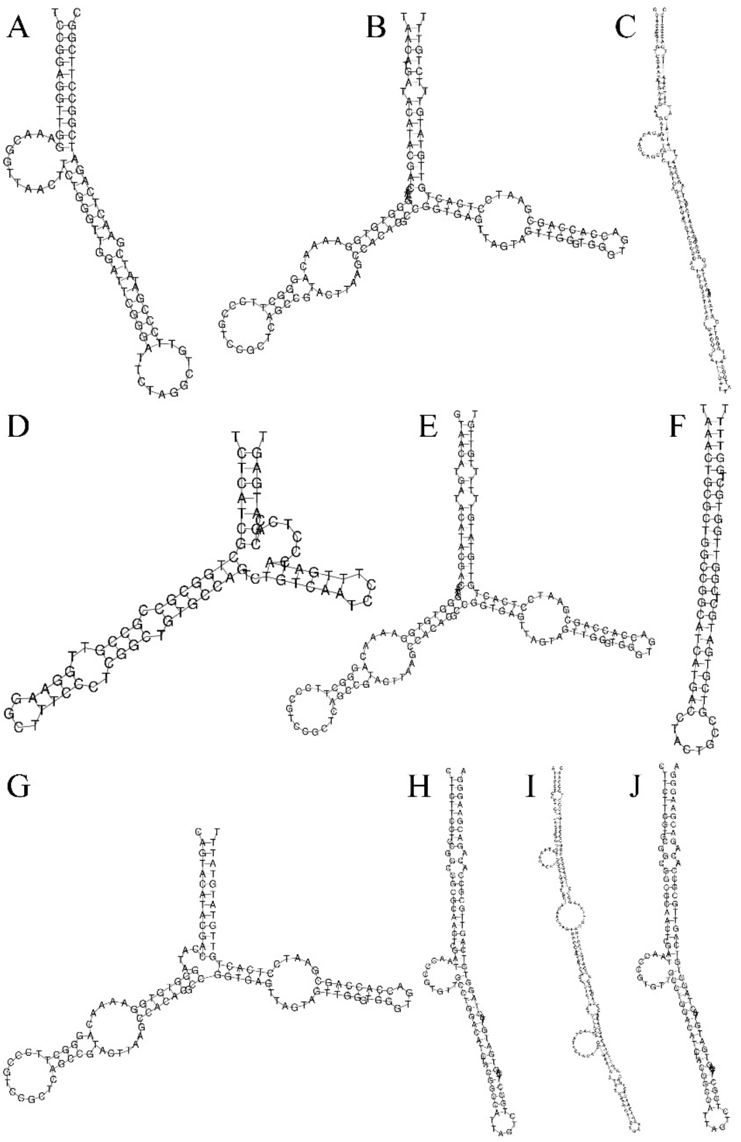


Figure S8. Stem-loop structure of novel miRNA precursor in *P. expansum.* A to J represent the novel miRNA 1 to novel miRNA 10.

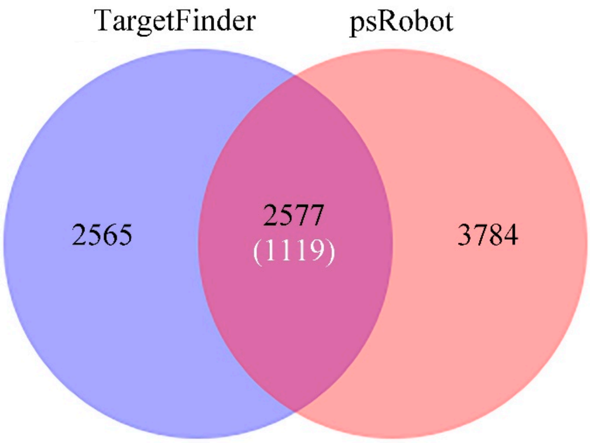


Figure S9. Venn statistics of miRNA target gene predicted by TargetFinder and psRobot software

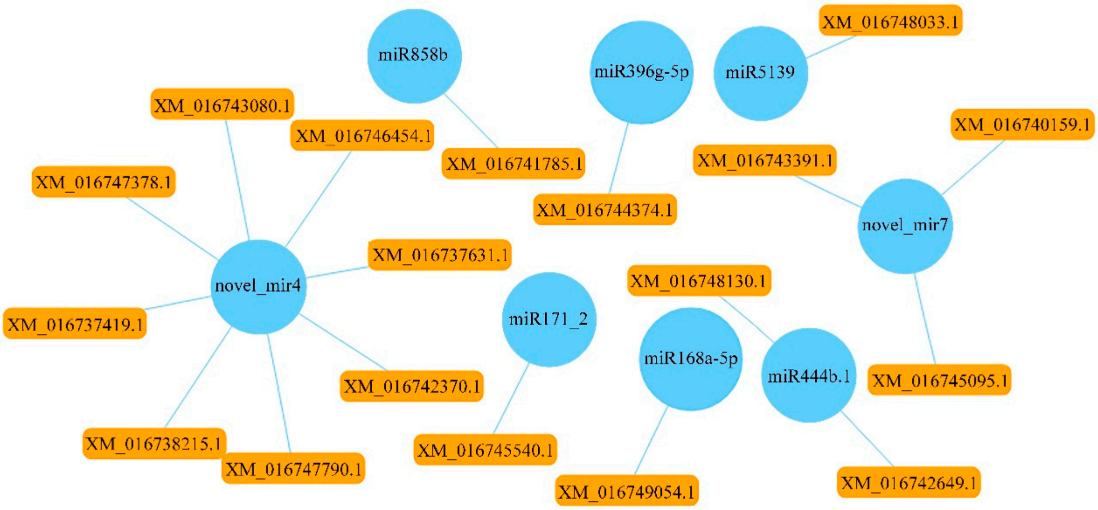


Figure S10. Interaction networks of DE miRNAs and DE mRNAs between Pe6h and P18h when the absolute value of Pearson correlation coefficient is higher than 0.6. The blue circles represent the DE miRNAs, and the rectangles with round corners represent the DE mRNAs.

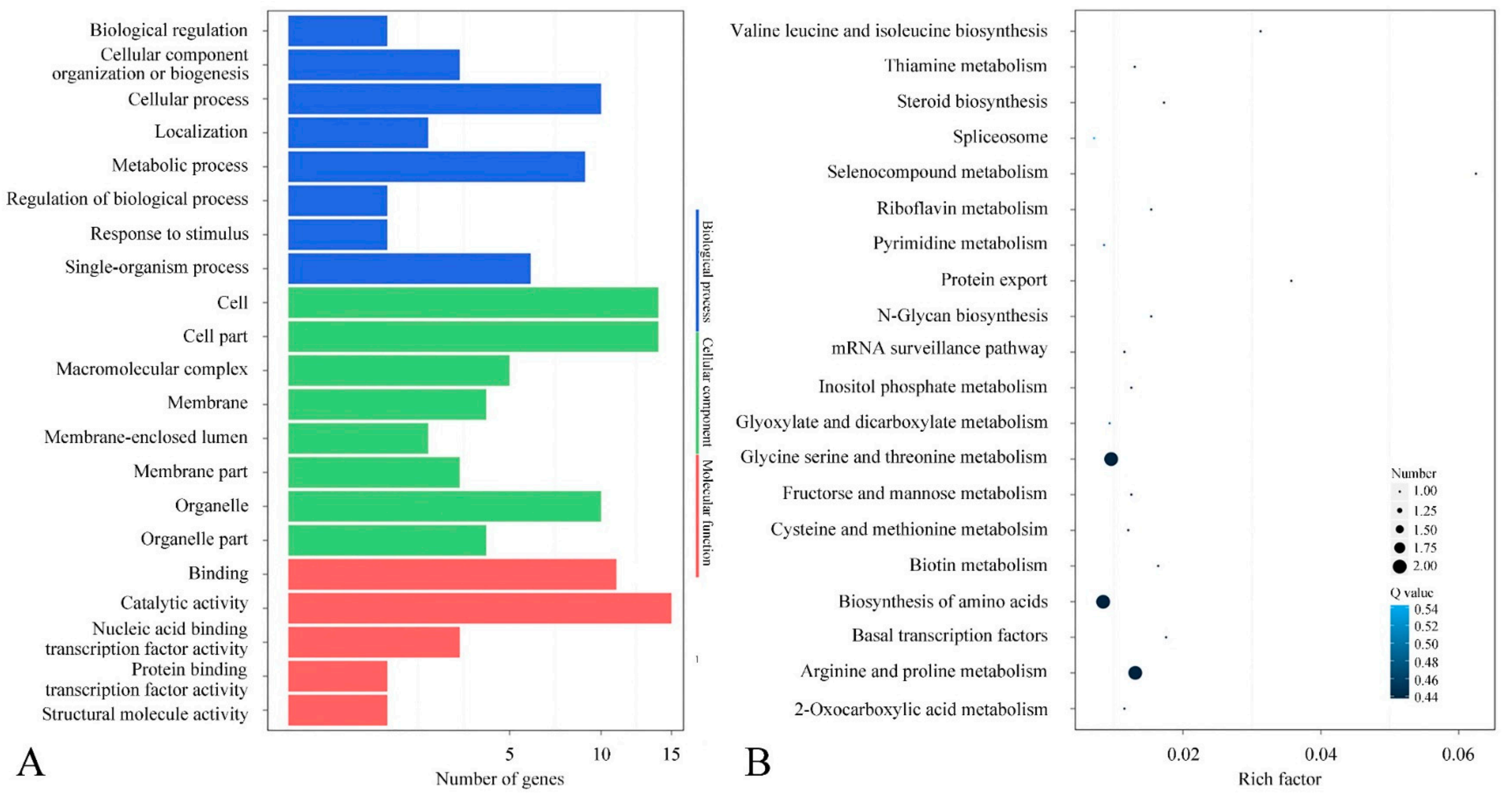


Figure S11. GO and KEGG enrichment analysis of DE miRNA target genes between Pe6h and Pe18h. These target genes are differentially expressed Pe6h and Pe18h as well; meanwhile, the expression correlation between DE miRNAs and their target genes is confirmed. A: GO

enrichment results. Different color represents different GO classification; B: KEGG enrichment results. The deeper color indicates the highest confidence. The rich factor is the ratio of the number of differentially expressed genes and the total identified genes in the same KEGG. The dot size indicated the number of DEGs.

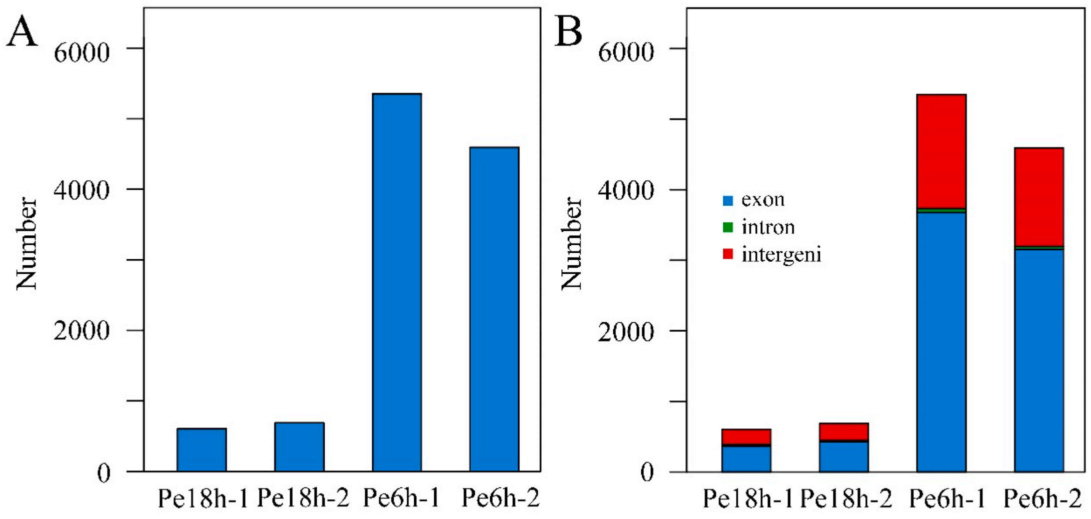


Figure S12. The statistics of circRNA number (A) and circRNA types (B) in each sample
